# Characterization and Control of *Thielaviopsis punctulata* on Date Palm in Saudi Arabia

**DOI:** 10.3390/plants11030250

**Published:** 2022-01-18

**Authors:** Khalid A. Alhudaib, Sherif M. El-Ganainy, Mustafa I. Almaghasla, Muhammad N. Sattar

**Affiliations:** 1 Department of Arid Land Agriculture, College of Agriculture and Food Sciences, King Faisal University, P.O. Box 420, Al-Ahsa 31982, Saudi Arabia; kalhudaib@kfu.edu.sa (K.A.A.); salganainy@kfu.edu.sa (S.M.E.-G.); malmghasla@kfu.edu.sa (M.I.A.); 2 Plant Pests, and Diseases Unit, College of Agriculture and Food Sciences, King Faisal University, P.O. Box 420, Al-Ahsa 31982, Saudi Arabia; 3 Vegetable Diseases Research Department, Plant Pathology Research Institute, ARC, Giza 12619, Egypt; 4 Central Laboratories, King Faisal University, P.O. Box 420, Al-Ahsa 31982, Saudi Arabia

**Keywords:** *Thielaviopsis punctulata*, date palm, black scorch disease, multi-locus phylogeny

## Abstract

Date palm (*Phoenix dactylifera* L.) is the most important edible fruit crop in Saudi Arabia. Date palm cultivation and productivity are severely affected by various fungal diseases in date palm-producing countries. In recent years, black scorch disease has emerged as a devastating disease affecting date palm cultivation in the Arabian Peninsula. In the current survey, leaves and root samples were collected from deteriorated date palm trees showing variable symptoms of neck bending, leaf drying, tissue necrosis, wilting, and mortality of the entire tree in the Al-Ahsa region of Saudi Arabia. During microscopic examination, the fungus isolates growing on potato dextrose agar (PDA) media produced thick-walled chlamydospores and endoconidia. The morphological characterization confirmed the presence of *Thielaviopsis punctulata* in the date palm plant samples as the potential agent of black scorch disease. The results were further confirmed by polymerase chain reaction (PCR), sequencing, and phylogenetic dendrograms of partial regions of the ITS, TEF1-α, and β-tubulin genes. The nucleotide sequence comparison showed that the *T. punctulata* isolates were 99.9–100% identical to each other and to the *T. punctulata* isolate identified from Iraq-infecting date palm trees. The pathogenicity of the three selected *T. punctulata* isolates was also confirmed on date palm plants of Khalas cultivar. The morphological, molecular, and pathogenicity results confirmed that *T. punctulata* causes black scorch disease in symptomatic date palm plants in Saudi Arabia. Furthermore, seven commercially available fungicides were also tested for their potential efficacy to control black scorch disease. The in vitro application of the three fungicides Aliette, Score, and Tachigazole reduced the fungal growth zone by 86–100%, respectively, whereas the in vivo studies determined that the fungicides Aliette and Score significantly impeded the mycelial progression of *T. punctulata* with 40% and 73% efficiency, respectively. These fungicides can be used in integrated disease management (IDM) strategies to curb black scorch disease.

## 1. Introduction

Date palm (*Phoenix dactylifera* L.) is the most extensively cultivated fruit tree plant in extreme arid and semi-arid regions due to its socio-economic and significant nutritional value [1]. Saudi Arabia is ranked second in date palm production, with an area of 157,000 ha being dedicated to it and 1.1 million tons of dates produced annually [2]. In Saudi Arabia, approximately 450 date palm cultivars have been cultivated in different regions [3]. Soil-borne pathogenic fungi cause serious infections and adversely affect the date quality and production [4,5]. 

The genus *Thielaviopsis* (family *Ceratocystidaceae*) (former *Ceratocystis pro parte*) includes six species: *T. cerberus*, *T. punctulata*, *T. ethacetica*, *T. musarum*, *T. euricoi*, and *T. paradoxa* [6]. Previously, these species were collectively called Ceratocystis paradoxa complex [7] and have been re-assigned by de Beer et al. [6] into the genus *Thielaviopsis* (type species *T. ethacetica*). Among them, *T. paradoxa* (anamorph of *C. paradoxa*) and *T. punctulata* (anamorph of *C. radicicola*) have been reported to be the causal agents of neck bending, wilting, black scorch, rhizosis, and chlorosis in young leaves not only in the Arabian Peninsula but also in other major date palm-producing countries [8,9]. These fungi infect a wide range of host plants, including coconut, palms, pineapple, and sugarcane [10,11]. Naturally, both species are soil-borne wound pathogens, which can confront any part of the date palm trees at any stage during their life cycle. Black scorch disease can cause economic losses to the date palm industry and may result in losses of newly planted off-shoots of ~50% [4]. This disease has a wide ecological distribution, with *T. paradoxa* being the major disease-causing agent on date palm plantations in Iran [12], Iraq [13], Italy [14], Kuwait [15], Oman [8], Qatar [16], Saudi Arabia [17,18], and the United States [19]. Moreover, *T. punctulata* has also been found to cause black scorch infestation on date palm trees in Oman [20], Qatar [16], and more recently, in the United Arab Emirates [5]. 

Once they have penetrated any vegetative part of the plant, these fungi cause severe rotting to occur in the buds, heart, inflorescence, leaves, and/or trunk of the plant [8]. Symptoms are characterized by tissue necrosis, the appearance of charcoal-like black and hard tissue lesions, the bending of the terminal bud or heart, and ultimately, sudden decline of the whole tree. Both *Thielaviopsis* species have been found to cause black scorch disease either independently or in combination with secondary fungal pathogens, such as *Alternaria* and *Phoma* spp. [8]. The disease severity may be exacerbated during abiotic stresses or due to poor horticultural practices [21].

The application of chemical control methods has been a major strategy to control black scorch disease despite the broad-spectrum negative impact of fungicides on the surrounding environment as well as their effects on human welfare. It has been found that applying difenoconazole provides effective control against black scorch disease in date palm plants [5]. Nevertheless, adopting integrated disease management (IDM) approaches may enormously reduce the use of chemicals and fungicides in crop plants [22,23]. The use of traditional management practices is also common to control black scorch disease. These may include avoiding wounds, cutting off diseased plant parts or the whole tree, and the integrated use of irrigation and fertilization [24,25]. However, an alternative to fungicides is the use of biological control agents (BCAs), which can provide fair control over the fungal population and can suppress their activity in plant tissues [26]. Thus, exploring native BCAs and/or their natural antagonistic products can also be a potential substitute for conventional fungicides to curtail black scorch disease. However, as a part of the long-term IDM approach, more recent genome editing and biotechnological approaches can provide fair control and can target fungal pathogens in date palm [27,28].

During a survey in the Al-Ahsa region, we observed withering, rhizosis (rapid decline), leaf wilting, neck bending, the yellowing of young leaves, and root necrosis in date palm plantations. The most frequently isolated pathogen from both necrotic roots and symptomatic leaves was *T. punctulata*. However, an extensive survey followed by the complete morphological, biological, and molecular characterization of the causal organism was required. Thus, this study was designed to study black scorch disease in date palm. The outcome of the proposed research project broadens our knowledge of the economic impact that *T. punctulata* has in the agro-ecological regions of Saudi Arabia. The study further explored the potential of various commercially available fungicides to develop an effective IDM strategy against *T. punctulata* in date palm.

## 2. Results

**Figure 1:**
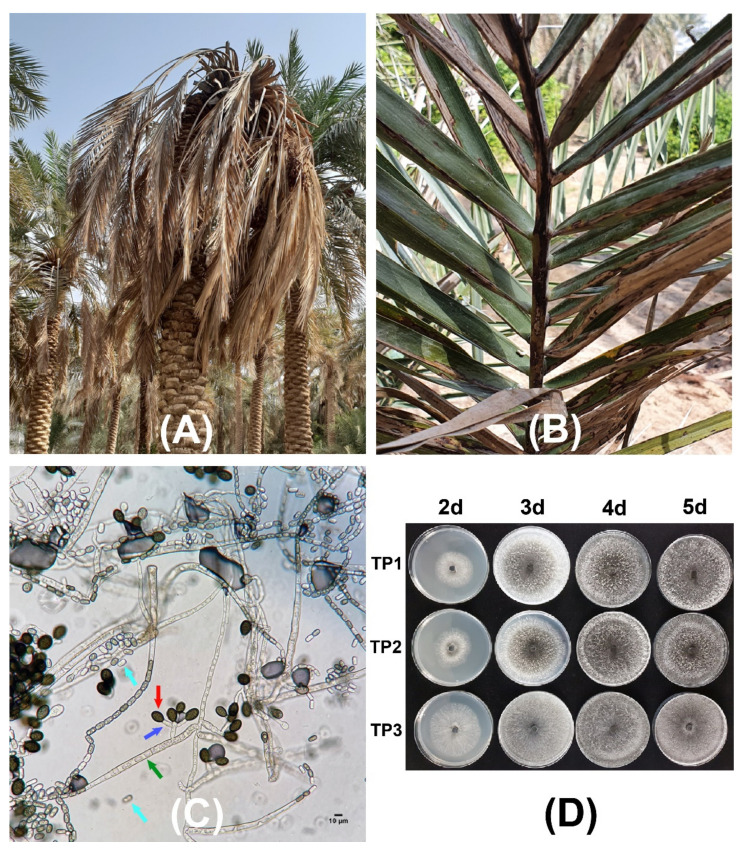
Black scorch disease symptoms and microscopic confirmation of the causal agent *Thielaviopsis punctulata*. Date palm plants showing neck bending (**A**,**B**) leaf drying under natural field conditions. (**C**) Mycelial growth of *T. punctulata* and conidiogenous cells showing aleuroconidia and phialoconidia. The arrows in green, red, blue, and turquoise represent *T. punctulata* septate hyphae, aleuroconidia, conidiophores, and phialoconidia, respectively. (**D**) Colony morphology of the three *T. punctulata* isolates (TP1, TP2, and TP3) after 2, 3, 4, and 5 days, at 21 °C on potato dextrose agar (PDA), respectively.

Date palm trees with progressed fungal infection and that were showing typical black scorch disease symptoms were found in the eastern region of Saudi Arabia. Different parts of the infected date palm trees were affected by the pathogen infection. The plant leaves developed a black charcoal-like hard appearance (Figure 1), while terminal bud infection resulted in severe head bending and in the complete drying of the infected tree (Figure 1A). During the survey of specific fields, entire date palm plants were observed to have black scorch disease symptoms. Other symptoms associated with the black scorch disease that were observed in the date palms were the drying of all of the leaves and, ultimately, absolute destruction of plants during the later stage of disease development (Figure 1). Three root samples (TP1, TP2, and TP3) were collected from three symptomatic date palm trees and were transferred to the laboratory for the isolation and characterization of the potential causal agents.

### 2.1. Morphological Characterization of Thielaviopsis punctulata as Causing Agent of Black Scorch Disease

**Figure 2:**
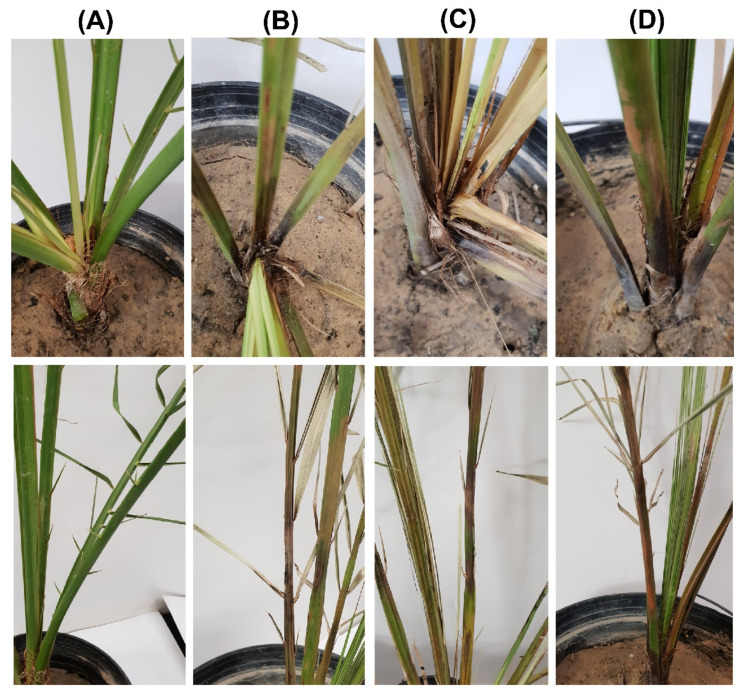
Pathogenicity test to fulfil Koch’s postulates for three *T. punctulata* isolates on date palm plants of the Khalas cultivar. The plants were inoculated with (**A**) double distilled water (control) and the *T. punctulata* isolates (**B**) TP1, (**C**) TP2, (**D**) and TP3, respectively. Typical symptoms as they appear on the heart (**upper panel**) and rachis (**lower panel**) are shown.

The microscopic examination showed optimum mycelial growth and the formation of two types of spores: aleuroconidia or chlamydospores and phialoconidia or endoconidial spores (Figure 1C,D). The thick-walled aleuroconidia were light to dark brown in color, oval-shaped, and were borne singly on the top of short hyphae, whereas the phialoconidia were hyaline to pale brown in color, were cylindrical-shaped, and were formed lengthwise in chains (Figure 1C). The dimensions of the aleuroconidia and phialoconidia were also measured to be 17.0 ± 1.73 × 10.0 ± 1.13 and 8.0 ± 1.01 × 4.0 ± 0.82 µm in size, respectively. The microscopic assessment of the morphology of 34 fungal isolates was suggestive of the presence of *T. punctulata* as the causal agent of black scorch in the infected samples. Finally, one purified fungal isolate from each sample (TP1, TP2, and TP3) was selected to perform pathogenicity tests by inoculating the tissue-cultured greenhouse-grown date palm plants (Figure 2). The inoculated plants developed black scorch disease symptoms, which initially presented as the browning of the stem tissues and branches, especially surrounding the inoculation site within 4 weeks of post inoculation (WPI). Later, as the infection progressed, the plants started showing black scorched leaves with leaf malformations, partial or complete tissue necrosis, and wilting at 6 WPI (Figure 2). To establish Koch’s postulates, the pathogen was re-isolated from the symptomatic tissues of date palm plants, and from the morphological data, it was confirmed that the pathogen was *T. punctulata*. After 6 WPI, all of the inoculated plants were severely discolored, and severe infections were observed on each part of the plants. However, the control plants did not show any symptoms.

### 2.2. Molecular Characterization of Thielaviopsis punctulata

The successful polymerase chain reaction (PCR) amplification of the three genes *internal transcribed spacer* (ITS) region of the nuclear ribosomal DNA (rDNA), *partial β-tubulin*, and *partial transcription elongation factor* 1-α (TEF1-α) from all of the date palm samples confirmed that the black scorch disease-causing *T. punctulata* fungi was frequently found in all samples.

**Figure 3:**
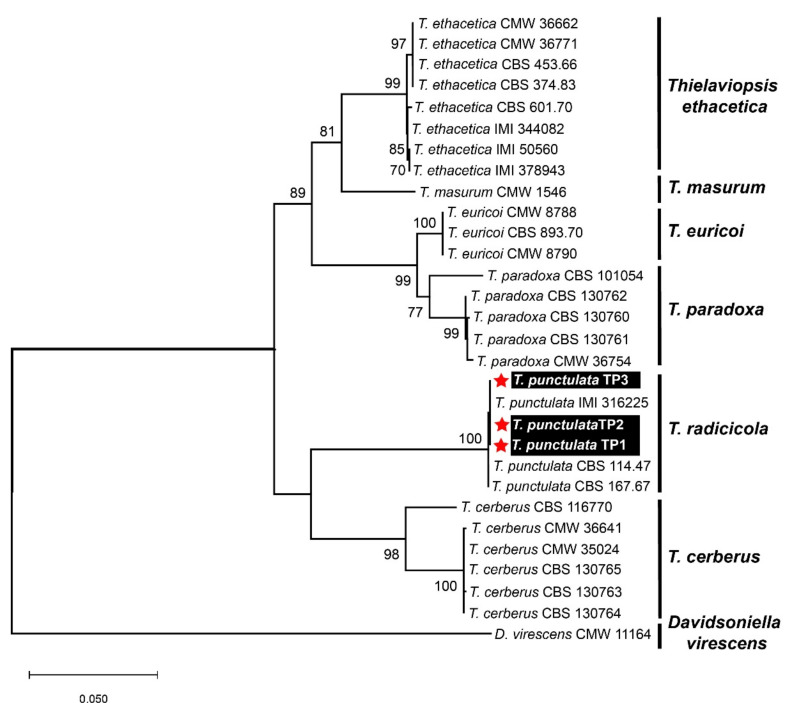
Molecular identification of three *Thielaviopsis punctulata* isolates based upon nucleotide sequences from the internal transcribed spacer (ITS), β-tubulin, and transcription elongation factor 1-α region from the selected isolates of different *Thielaviopsis* species. A maximum-likelihood (ML) algorithm was employed to construct a combined phylogenetic dendrogram by combining the ITS, β-tubulin, and TEF1-α sequences using the best-fit Kimura 2-parameter model (T93+G) in MEGA X software. The isolates that were identified from Saudi Arabia in this study are highlighted in white text on black background and with red asterisks. Only bootstrap values (numeric values at the branch nodes) ≥70 were shown to support the evolutionary relatedness of the isolates. The descriptors for all fungal isolates were named according to de Beer et al. [6].

No DNA sequence data for the *T. punctulata* species are available from Saudi Arabia in the GenBank and/or in the available literature. Therefore, to ensure sequence relatedness and the evolutionary relationships of these *T. punctulata* isolates to other *Thielaviopsis* spp., a detailed sequence comparison was performed, and a phylogenetic dendrogram was constructed, including the available nucleotide (nt) sequences of the aforementioned three genes, which represent the genes that are most closely related to *Thielaviopsis* spp. (Table 1, Figure 3). The nt sequences of the *ITS*, *β-tubulin*, and *TEF1-α* genes from the three isolates were deposited in the GenBank to acquire their respective accession numbers: MZ701784-MZ701786, MZ703651-MZ703653, and MZ703648-MZ703650, respectively (Table 1). A combined phylogenetic dendrogram was constructed for all of the isolates, and it was confirmed that the three isolates in our study were grouped into a well-supported clade (100% bootstrap value) with the *T. punctulata* isolates that had been reported from Iraq, Mauritania, and the United States (Figure 3). The nt sequence comparison showed that our isolates shared 99.9–100% nt sequence identities with each other and with the *T. punctulata* isolate (IMI 316225) identified from the infected date palm trees in Iraq. Whereas, with the other two *T. punctulata* isolates reported from the United States (CBS 114.47) and Mauritania (CBS 167.67), our isolates shared nt sequence identities that were 99.8 and 99.7–99.8% similar, respectively. The nt sequence comparisons and the phylogenetic dendrogram supported that the fungal isolates that were identified from Saudi Arabia were members of the *T. punctulata* species.

### 2.3. In Vitro and In Vivo Growth Inhibition Efficiency of Fungicides against Thielaviopsis punctulata

**Figure 4:**
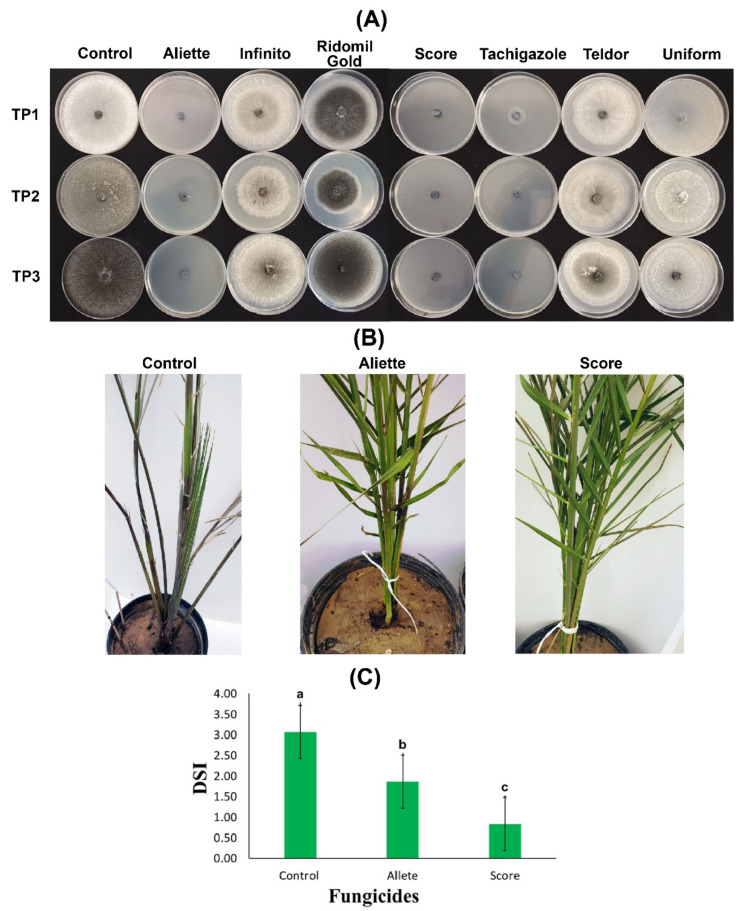
In vitro and in vivo evaluation of fungicides against *Thielaviopsis punctulata*. (**A**) Percentage growth inhibition of mycelium from eight fungicides: Aliette, Infinito, Ridomil Gold, Score, Tachigazole, Teldor, and Uniform. The efficiency of each fungicide was tested on potato dextrose agar (PDA) media against three *T. punctulata* isolates: TP1, TP2, and TP3. (**B**) Date palm plants of the Khalas cultivar were first inoculated with the *T. punctulata* isolate TP2, and at 2 WPI, the plants were treated with the fungicides Aliette and Score. The healthy control was mock-inoculated using double distilled water. (**C**) Disease severity index (DSI) of the infected date palm plants at 8 WPI treated with the Aliette and Score fungicides. The significant values at *p* > 0.05 are shown as different lowercase letters.

The seven tested fungicides showed variable efficiency to curb the mycelial growth of the three *T. punctulata* isolates. The data showed that there were significant differences among all of the fungicides in their ability to inhibit *T. punctulata* mycelium growth (Table 2). The results further revealed that Aliette, Score, and Tachigazole successfully prevented the growth and sporulation of the three isolates. The results were further confirmed when mycelium growth inhibition was measured. A clear fungal inhibition zone was observed (~100%) when the potato dextrose agar (PDA) media was supplemented with Aliette, Score, and Tachigazole. An exception was observed in the case of the TP1 isolate when Tachigazole was applied with a fungal inhibition zone >86%. There was a significant reduction in the mycelium inhibition zone, which showed an 8 to 24% reduction with the supplementation of the Telder fungicide, whereas no significant difference was observed between Ridomil, Gold, and Uniform because virtually no mycelium growth or sporulation inhibition were observed (Table 2, and Figure 4A).

To further confirm our in vitro results, two fungicides, Aliette and Score, were sprayed on the date palm Khalas cultivar plants infected with the TP2 isolate of *T. punctulata* under greenhouse conditions, and the efficiency of both fungicides was observed for 8 WPI. Before fungicide spraying, the plants started showing black scorch symptoms at 2 WPI. However, in the plants treated with Score at 4 WPI, the disease progression was significantly reduced, and the fungicide efficiency was 73% compared to the control at 8 WPI (Figure 4B). Although the disease progression was reduced with 40% efficiency with Aliette, it was not a significant reduction compared to the reduction observed for Score. The date palm plants treated with the Score fungicide also showed the emergence of new leaves from the heart. Contrarily, the untreated plants (control) started to dry. Moreover, the disease severity index (DSI) was drastically reduced to 0.83 and 1.87 at 8 WPI compared to 3.07 in the control plants (Figure 4C). Thus, the results of the Score fungicide application were highly promising against all three isolates of *T. punctulata* in our study.

## 3. Discussion

The date palm has been traditionally cultivated for over 5000 years in arid and semi-arid regions not only because of its edible significance but also due to its social and cultural value [29]. Saudi Arabia contributes ~9 million tons to the global date production with a share of about 17% (https://www.fao.org/faostat/en/#data/QC last accessed 10 December 2021). Among the major biotic stresses, diseases caused by soil-borne fungal pathogens are a major menace to date palm production in Saudi Arabia and include leaf spot, leaf blight, sudden decline, and black scorch disease [18]. In Saudi Arabia, *Fusarium proliferatum*, *F. solani*, *F. brachygibbosum*, *F. oxysporum* and *F. verticillioides* have been recently characterized as causing different fungal-associated diseases [30,31]. Black scorch is another fungal disease that can affect date palm cultivation and is caused by *T. paradoxa* and *T. punctulata* in different parts of the world. Likewise, in Saudi Arabia, both *T. paradoxa* and *T. punctulata* have been found to be associated with black scorch disease in date palm [17,18,32]. However, these studies only focused on the morphological identification of the fungal pathogens and could not determine the molecular anomalies of *Thielaviopsis* spp. in Saudi Arabia. In field surveys, it is difficult to discriminate between black scorch and sudden decline disease due to their similar symptoms. Thus, PCR-based molecular diagnostic techniques can help to differentiate between sudden decline and black scorch disease and can aid in the development of effective control strategies [33]. Black scorch has not been established as an epidemic to date in palm cultivation; however, if established, it may lead to heavy losses in date palm cultivation. Therefore, comprehensive research investigating this important date palm disease is crucial. With this in mind, the current study was designed to pinpoint the exact causal agent of black scorch disease in Saudi Arabia and aimed to find a potential solution to control this devastating pathogen.

The primary morphological data were suggestive of *T. punctulata* being a potential causal agent of the black scorch disease in the collected field samples. It was evident from the microscopic investigation that the pathogen produced abundant endoconidia in the PDA media. The physiological data were based on those acquired from previous studies in which thick-walled chlamydospores appeared singly on short hyphal branches and were light to dark brown in color. Moreover, we observed hyaline to pale brown phialoconidia formed in chains. The length and width of aleuroconidia and phialoconidia were measured as being 17.0 ± 1.73 × 10.0 ± 1.13 and 8.0 ± 1.01 × 4.0 ± 0.82 µm in size, respectively. Our results were in accordance with previous reports that aleuroconidia are often larger than phialoconidia in *Thielaviopsis* species [5,17,34]. Although it is difficult to identify fungal species based on spore morphology, it is crucial to study spore morphology because of its critical role in the dispersal, pathogenicity, and survival of the fungal pathogen under study [5]. To elucidate the nature and pathogenicity of the potential pathogen, we inoculated tissue-cultured greenhouse-grown date palm plants with the three isolates of the pathogen. Our data were very similar to the data obtained from previous pathogenicity studies of *T. paradoxa* on *Dracaena marginata* [35], *Butia capitate* [36], *Hyophorbe lagenicaulis* [37], and *Cocos nucifera* [38]. Hence, based upon the morphology and sporulation characteristics, it is difficult to distinguish both *Thielaviopsis* spp., and a DNA-based molecular characterization of the most conserved regions of these fungal pathogens can be an alternative species-specific detection method for black scorch disease in date palm.

Based upon the previous literature, specific genomic regions corresponding to the *ITS*, *β-tubulin*, and *TEF1-α* genes were PCR-amplified and were subsequently sequenced. A combined phylogenetic dendrogram and nt comparison of the three corresponding genes from different *Thielaviopsis* spp. showed that our three isolates were the most closely related to the *T. punctulata* isolate IMI 316225 (nt sequence identity 100%) identified in Iraq. Although the nt sequence identity was shown to be 99.7–99.8% with two other *T. punctulata* isolates CBS 114.47 and CBS 167.67 from the USA and Mauritania, all of the isolates showed a similar evolutionary lineage, as evident from the phylogenetic dendrogram (Figure 3). None of the isolates were grouped with the *T. paradoxa* in the phylogenetic analysis. Thus, based upon our detailed investigation and in contrast to previous morphological studies [17,18,32], we may speculate that the causal agent of black scorch disease in Saudi Arabia is *T. punctulata* and not *T. paradoxa*. Our conclusion is also supported by other reports from the Arabian Peninsula, where it has been shown that *T. punctulata* is the black scorch disease-causing agent in this region [5,8,16,39]. However, an extensive survey across the major date palm-producing areas in the country can better answer this speculation. Molecular monitoring of the soil-borne fungal pathogens has become an important management tool [40]. Thus, application of a high-throughput, precise, and more sensitive molecular diagnostic method to discriminate between *T. punctulata* and *T. paradoxa* species can help to devise species-specific control strategies in date palm. Furthermore, we extended our investigation to find a better control strategy to circumscribe black scorch disease in date palm.

Over-reliance on chemical-based pesticides and fungicides is hazardous to an agro-ecological ecosystem and may result in the development of pathogen resistance [23]. However, studies on the integrated reduced use of conventional fungicides against *T. punctulata* are very limited [5,25]. Therefore, we continued our work through both *in vitro* and in vivo studies to determine the fungicide that is the most efficient in providing stable control against *T. punctulata* in Saudi Arabia. We selected seven systemic fungicides (Aliette, Infinito, Ridomil Gold, Score, Tachigazole, Teldor, and Uniform) to test their efficacy against black scorch disease. The in vitro investigation using PDA showed that Aliette and Score were the most efficient fungicides at the tested concentration of 300 ppm, providing 100% mycelium inhibition against the three tested *T. punctulata* isolates (Figure 4A), whereas Tachigazole showed 87–100% mycelium inhibition against the three *T. punctulata* isolates. The rest of the fungicides Infinito, Ridomil Gold, Teldor, and Uniform could not produce promising results against the mycelial growth of *T. punctulata*. Henceforth, Aliette (Fosetyl-Al) and Score (Difenoconazole) appear to be the best candidate fungicides for the control of *T. punctulata* in date palm followed by Tachigazole (Hymexazol). However, an empirical demonstration was necessary to test their efficacy against *T. punctulata* under controlled environmental conditions. Our in vivo results on date palm seedlings showed that Score was the most efficient fungicide against *T. punctulata* with ~73% efficiency and 0.83 DSI compared to Aliette, with nearly 40% efficiency and 1.87 DSI against black scorch disease (Figure 4B,C). In a previous study, Croft [41] showed that difenoconazole (Score) was unable to accelerate sugarcane seed germination with *T. paradoxa*. In contrast, our results were similar to the findings of Saeed et al. [5], who found that the difenoconazole-based Score fungicide showed >91% mycelium inhibition against *T. punctulata* in PDA media. In the same study, Saeed et al. [5] reported highly significant inhibition of *T. punctulata* in inoculated date palm seedlings.

## 4. Materials and Methods

### 4.1. Collection of Symptomatic Date Palm Plant Samples

The root samples were collected from symptomatic date palm trees in three different locations in the Al-Ahsa province of Saudi Arabia (Figure 1A,B). The collected root samples were washed with tap water to remove any soil particles that had adhered to the roots. The clean roots were fused for further fungal isolation and characterization.

### 4.2. Isolation of the Root Rot Fungal Pathogens

Tissues were excised from the infected lesions or roots, surface-sterilized using 2% sodium hypochlorite for 2 min followed by rinsing two times with sterilized distilled water, and were dried between pieces of filter paper for 10 min at room temperature. The clean and sterilized pieces of each sample were overlaid on five PDA plates supplemented with 100 ppm ampicillin to avoid any bacterial contamination. Petri plates were incubated at 27 °C for 3–5 days in complete darkness. At least 2–3 emerging fungal colonies were individually subcultured from each plate on PDA for further identification. The morphology of the mycelium and spores was observed under a light microscope (Leica, DM 25000 LED) to characterize different mycological structures (Figure 1C). The spore dimensions were measured using Leica application suite X (LasX). Photographs were taken using a Flexacam C1 camera.

### 4.3. Pathogenicity Test and Disease Assays

The virulence of the selected isolates was performed to determine their aggressiveness on tissue-cultured plants of the date palm cultivar Khalas grown in soil pots according to the method used by Saeed et al. [5]. After the plant surface had been sterilized with 70% ethanol, the wounded parts of the plants were sprayed with a 5 × 10^5^ mL^−1^ spore suspension of *T. punctulata* (wounds were made with a sterile needle) at the leaf base of the plants to facilitate infection. Moreover, the soil pots of the same plants were also infested with *T. punctulata* by drenching 50 mL of potato broth medium (PBM) with 1 × 10^6^ mL^−1^ spore suspension. The control plants were only sprayed with sterilized distilled water, and soil was drenched by 50 mL of PBM only. Plastic bags were used to cover the inoculated plants to provide sufficient humidity for infection, and the samples kept at 27 °C under controlled environmental conditions. The plants were examined closely for any black scorch disease symptoms for 6 weeks. The disease severity index was calculated using the scale from 0 to 5, as previously described [5,32].

### 4.4. Molecular Characterization of the Purified Isolates of Thielaviopsis punctulata

Total genomic DNA was extracted from the dried mycelium that had been cultured on PDA media following the Dellaporta extraction method [42], with some minor modifications. The extracted DNA was used either directly for a PCR or was frozen for further experiments. The PCR reactions were performed to amplify the ITS region, partial β-tubulin, and partial TEF1-α using their respective primers (Table 3). PCR amplification was carried out as previously described [7] using the ESCO Swift Maxi Thermal Cycler. In a 25 µL total reaction volume, ~40 ng of fungal DNA were mixed with 2.5 µL of 10 × Taq Polymerase buffer, 2 mM MgCl2, 1.5 µL of 10 µM primers, 2.5 µL of 10 mM dNTPs, 0.3 µL of 5U Taq DNA Polymerase, and a final reaction volume with nuclease-free water. The PCR was performed as initial denaturation at 95 °C for 2 min, followed by 35 cycles of 95 °C for 30 s, 52–58 °C for 30–60 s, and 72 °C for 30 s, and the final elongation cycle performed at 72 °C for 10 min. The resultant PCR amplicons were further confirmed by agarose gel electrophoresis. The confirmed PCR products were purified using the CloneJet PCR cloning kit (ThermoFisher Scientific, Waltham, MA, USA) and were completely sequenced at Macrogen Inc., (Seoul, Korea).

The obtained sequences for ITS, β-tubulin, and TEF1-α were initially compared to their respective sequences using BLASTn in the NCBI GenBank database for their primary identification. The highly similar sequences for each region were retrieved from the NCBI. The sequences of each gene in the study were aligned separately with the respective sequences of the most closely related *Thielaviopsis* spp. isolates using the ClustalW algorithm in MEGA X [46]. Thus, three individual datasets including sequences from each and the relevant sequences retrieved from the NCBI were constructed. Finally, a combined dataset of all three genes was generated to construct a phylogenetic dendrogram and to infer the evolutionary relationships. Furthermore, the pairwise nt sequence identities of all of the fungal isolates were calculated using the Muscle algorithm that is available in the species demarcation tool (SDTv1.2) software (University of Cape Town, Cape Town, South Africa).

### 4.5. In Vitro Evaluation of Fungicides against Thielaviopsis punctulata

The antifungal evaluation of seven fungicides: Aliette (80% WG), Infinito (687.5 SC), Ridomil Gold (480 SL), Score (250 EC), Tachigazole (30% SL), Teldor (50 SC), and Uniform (446 SE), was performed as previously described [47] (Table 4). The fungicide solution of each fungicide contained a 300 ppm final concentration of each respective fungicide. These fungicide solutions were then aseptically introduced into the sterilized PDA media supplemented with ampicillin at 55 °C to avoid any bacterial growth. The media was carefully poured into the Petri plates. The fungal plug ~5 mm of each *T. punctulata* isolate was transferred to the control and the treatment plates followed by incubation at 25 °C for six days according to the method used by Jonathan et al. [47]. After six days, the radial growth of each fungal isolate was measured in the dishes to determine the growth inhibition efficiency of each fungicide. The percentage mycelium growth inhibition was measured as follows:

MI% = 100 × (Mc − Mt)/Mc

where Mc is the diameter of the mycelium growth on the medium without fungicide, while Mt is mycelium growth on the medium with each fungicide.

### 4.6. In Vivo Evaluation of Fungicides to Control Black Scorch Disease

Two-year-old tissue-cultured plants from the date palm cultivar Khalas grown under greenhouse conditions were used to assess the efficacy of two fungicides, i.e., Aliette and Score, against black scorch disease. The plants were inoculated at the leaf base region with *T. punctulata* isolate TP2, as described above. The inoculated plants were kept in greenhouse conditions and were covered with transparent plastic bags for 5 days to facilitate fungal infection. After 2 WPI, the plants were sprayed with the commercially recommended doses of the respective fungicides. The control plants were only sprayed with double distilled water. The experiment was repeated three times with five replicates for each treatment.

### 4.7. Data Analysis

The data for the in vitro experiments were analyzed using a two-way analysis of variance (ANOVA) approach with a treatment effect using the general linear model (GLM) procedure. The least significant difference (LSD) test was applied to separate the means at the 5% significance level. 

The in vivo evaluation of two fungicides on the infected date palm plants was performed in a completely randomized design (CRD) under greenhouse conditions. For the disease assay and in vivo assessment of the Aliette and Score fungicides, three replicates with five plants per replicate were used. The significance was determined using the LSD test with the statistical difference set at *p* < 0.05. The collected data were statistically analyzed using the MSTAT-C program (v 2.10). All statistical analyses were carried out using SAS/STAT^®^ 9.3 software (SAS, Cary, NC, USA)

## 5. Conclusions

Our study confirmed *T. punctulata* as the black scorch disease-causing agent in Saudi Arabia. Although black scorch disease has already been reported from Saudi Arabia, previous investigations were based upon morphological characterizations of the pathogen. Our research represents a detailed study on the morphology, pathogenicity, biology, evolutionary relatedness, and potential control mechanisms of *T. punctulata* in Saudi Arabia. However, further research should explore the complete infection cycle of *T. punctulata* and the development of an effective IDM approach.

## Figures and Tables

**Table 1:** List of *Thielaviopsis* spp. for which isolates or sequences were included in this study.

Species Name	* Isolate	GenBank Accession Numbers			Host	Origin
		ITS	β-Tubulin	TEF1-α		
*Thielaviopsis cerberus*	CBS 130763	JX518355	JX518387	JX518323	*Theobroma cacao*	Cameroon
	CMW 35024	JX518356	JX518388	JX518324	*T. cacao*	Cameroon
	CMW 36641	JX518345	JX518377	JX518313	*Elaeis guineensis*	Cameroon
	CBS 130764	JX518349	JX518381	JX518317	*E. guineensis*	Cameroon
	CBS 130765	JX518348	JX518380	JX518316	*E. guineensis*	Cameroon
*T. ethacetica*	CBS 374.83	JX518329	JX518361	JX518297	*Phoenix canariensis*	Spain
	CBS 601.70	JX518331	JX518363	JX518299	*Ananas comosus*	Brazil
	CBS 453.66	JX518332	JX518364	JX518300	*Cocos nucifera*	Nigeria
	CMW 36662	JX518353	JX518385	JX518321	*E. guineensis*	Cameroon
	CMW 36771	JX518330	JX518362	JX518298	*Saccharum* sp.	South Africa
	IMI 50560	JX518341	JX518373	JX518309	*A. comosus*	Malaysia
	IMI 344082	JX518339	JX518371	JX518307	*C. nucifera*	Tanzania
	IMI 378943	JX518340	JX518372	JX518308	*E. guineensis*	Papua New Guinea
*T. euricoi*	CMW 8788	JX518326	JX518358	JX518294	*C. nucifera*	Indonesia
	CMW 8790	JX518327	JX518359	JX518295	*C. nucifera*	Indonesia
	CBS 893.70	JX518335	JX518367	JX518303	*C. nucifera*	Brazil
*T. paradoxa.*	CBS 130760	JX518346	JX518378	JX518314	*E. guineensis*	Cameroon
	CBS 130762	JX518352	JX518384	JX518320	*E. guineensis*	Cameroon
	CBS 130761	JX518342	JX518374	JX518310	*T. cacao*	Cameroon
	CMW 36754	JX518344	JX518376	JX518312	*E. guineensis*	Cameroon
	CBS 101054	JX518333	JX518365	JX518301	*Rosa* sp.	Netherlands
	CBS 116770	JX518334	JX518366	JX518302	Palm sp.	Ecuador
*T. musarum*	CMW 1546	JX518325	JX518357	JX518293	*Musa* sp.	New Zealand
*T. punctulata*	CBS 114.47	KF612023	KF612025	KF612024	*P. dactylifera*	USA
	CBS 167.67	KF953932	KF953931	KF917202	*Lawsonia inermis*	Mauritania
	IMI 316225	JX518338	JX518370	JX518306	*P. dactylifera*	Iraq
	TP1	MZ701784	MZ703651	MZ703648	*P. dactylifera*	Saudi Arabia
	TP2	MZ701785	MZ703652	MZ703649		
	TP3	MZ701786	MZ703653	MZ703650		
*Davidsoniella virescens*	CMW 11164	AY528984	AY528990	AY528991	*Quercus* sp.	USA

* CBS: Centraalbureau voor Schimmelcultures. CMW: Culture collection of the Forestry, and Agricultural Biotechnology Institute (FABI), University of Pretoria. IMI: International Mycological Institute. TP1-TP3: Three *Thielaviopsis punctulata* isolates (bold text) identified in the current study.

**Table 2:** Percentage colony growth inhibition of three isolates of *Thielaviopsis punctulata* using seven fungicides.

Fungicide	TP1	TP2	TP3
Aliette- 80% WG	100 a	100 a	100 a
Infinito 687.5 SC	3.33 d	36.67 b	6.00 bc
Ridomil Gold- 480 SL	0.00 d	41.11 b	5.33 bc
Score 250 EC	100 a	100 a	100 a
Tachigazol- 30% SL	86.67 b	100 a	100 a
Teldor 50 SC	23.78 c	1.78 c	8.22 b
Uniform-446 SE	0.00 d	0.00 d	2.44 cd
Control	0.00 d	0.00 d	0.00 d

Values with similar letters show non-significant differences at *p* < 0.05.

**Table 3:** Nucleotide sequence of the primers used in the study.

Locus Name	Primer	Sequence (5′-3′)	PCR Program *	Reference
ITS region	ITS4	TCCTCCGCTTATTGATATGC	35 cycles at 94 °C for 30 s, 52 °C for 30 s, 72 °C for 30 s	[43]
	ITS5	GGAAGTAAAAGTCGTAACAAGG		
TEF1-α	EF1F	TGCGGTGGTATCGACAAGCGT	35 cycles at 94 °C for 30 s, 58 °C for 60 s, 72 °C for 90 s	[44]
	EF1R	AGCATGTTGTCGCCGTT GAAG		
β-tubulin	Bt1a	TTCCCCCGTCTCCACTTCTTCATG	34 cycles at 94 °C for 60 s, 58 °C for 60 s, 72 °C for 60 s	[45]
	Bt1b	GACGAGATCGTTCATGTTGAACTC		

* Initial denaturation at 95 °C for 2 min and the final elongation cycle performed at 72 °C for 10 min, respectively.

**Table 4:** List of fungicides and their characteristics used to curtail black scorch disease in date palm.

Fungicides	Active Ingredient	Chemical Group	Dose/L
Aliette- 80% WG	Fosetyl-Al	Organophosphate	2.5 g
INFINITO 687.5 SC	Fluopicolide + Propamocarb HCL	Acylpicolide + Carbamate	1.5 mL
RIDOMIL GOLD- 480 SL	Metalaxyl-M	Phenylamide	2 mL
Score 250 EC	Difenoconazole	Triazole	0.5
TACHIGAZOL- 30% SL	Hymexazol	Oxazoles	1.5 mL
Teldor 50 SC	Fenhexamid	Anilide	0.5 mL
UNIFORM-446 SE	Metalaxyl-M + Azoxystrobin	Phenylamide + Strobilurin	0.5 mL

## Data Availability

Not Applicable.
